# Is It Possible to Train the Endothelium?—A Narrative Literature Review

**DOI:** 10.3390/life14050616

**Published:** 2024-05-10

**Authors:** Karolina Biernat, Natalia Kuciel, Justyna Mazurek, Katarzyna Hap

**Affiliations:** University Rehabilitation Centre, Wroclaw Medical University, 50-367 Wroclaw, Poland; karolina.biernat@umw.edu.pl (K.B.); justyna.mazurek@umw.edu.pl (J.M.); katarzyna.hap@umw.edu.pl (K.H.)

**Keywords:** endothelium, exercise, cardiovascular system

## Abstract

This review provides an overview of current knowledge regarding the adaptive effects of physical training on the endothelium. The endothelium plays a crucial role in maintaining the health of vessel walls and regulating vascular tone, structure, and homeostasis. Regular exercise, known for its promotion of cardiovascular health, can enhance endothelial function through various mechanisms. The specific health benefits derived from exercise are contingent upon the type and intensity of physical training. The review examines current clinical evidence supporting exercise’s protective effects on the vascular endothelium and identifies potential therapeutic targets for endothelial dysfunction. There is an urgent need to develop preventive strategies and gain a deeper understanding of the distinct impacts of exercise on the endothelium.

## 1. Introduction

It is well known that regular physical activity induces beneficial physiological changes, significantly reducing the risk of obesity, type 2 diabetes, and cardiovascular diseases. Physical exercise is a fundamental element of a healthy lifestyle and an essential part of non-pharmacological therapy for many diseases, often determining their course and prognosis [1,2,3]. Physical training helps to maintain endothelial function and provides a robust protective effect against endothelial damage [4,5,6,7,8]. The benefits of exercise on endothelial function may be related to direct hemodynamic effects or secondary effects, such as the modification of risk factors [9]. Skeletal muscle consumes more oxygen and energy during physical exercise, which affects the heart rate, myocardial contractility, arterial pressure, blood flow, and vasodilation, which is dependent on the endothelium and affects the regulation of energy and oxygen demand [10]. During physical exercise, there is an increased production, bioavailability, and synthesis of nitric oxide (NO) in endothelial cells, which leads to the relaxation of the smooth muscles of blood vessels [11]. Increased NO production and hemodynamic changes result from accelerated blood flow during physical exercise—this causes endothelial cells to be exposed to shear forces and contributes to the modification of vascular function. The dilation of blood vessels during physical activity stems from the parallel action of nitric oxide and potassium ions released during the contraction of muscle fibers. The joint action of potassium ions and physical exercise on capillaries results in hyperemia and hyperpolarization of smooth muscles and vascular endothelial cells [12]. Many researchers and clinicians indicate the occurrence of endothelial dysfunction as the underlying cause of many diseases [13,14,15,16,17]. Impaired vasodilation is strongly associated with the development of cardiovascular disease and pathological changes toward vasoconstriction, thrombosis, and inflammation. Endothelial dysfunction is one of the first elements initiating the development of atherosclerosis, which can be effectively inhibited through early intervention with physical exercise [18,19,20].

**Aim** 
The purpose of this narrative review was to explore the adaptive effects of physical training on the endothelium.
**Methods** 
We use both quantitative and qualitative methods to screen papers and provide insights into the inclusion and exclusion criteria. We outline our approach for article selection and provide an overview of our findings. This is followed by a more detailed insight into selected articles. The research question guiding our analysis was: what is said about the impact of physical training on the endothelium?
Inclusion: This narrative review include pivotal or seminal papers that address the phenomenon of interest and other manuscripts that are relevant to the research question (experimental or observational, systematic reviews, meta-analyses). Excluded publications are editorials and letters and all articles that were published in a language other than English.
Search terms: endothelium, health, physical exercise, endothelial function, benefits, exercise training, dynamic exercise, endurance training, high intensity interval training, resistance training, isometric training, aerobic exercise, microvascular, patients.
Datebases: PubMed, Cochrane Library, Web of Science, and Scopus were screened against the eligibility criteria.
Time frame/years searched: between 1980—the first paper found on this topic—and 2024.
Date of search: 2 February 2024.
Results: 78 articles/full texts were included in the study according to the selection criteria.

## 2. Physiological Function of Vascular Endothelium

Since the groundbreaking work by Furchgott and Zawadzki, recognizing the endothelium as the main regulator of blood vessel homeostasis and identifying the main compound involved in the relaxation of vascular muscles, the awareness of its role in the body has increased significantly [21]. The endothelium of an individual weighing 70 kg is approximated to encompass an area of around 700 square meters and has a weight ranging from 1 to 1.5 kg [22,23]. The endothelium has many functions, including regulation of the tension of blood vessel walls, barrier function, regulation of blood circulation and pressure, maintaining the balance between the processes of blood coagulation and fibrinolysis, participation in the reactions of the immune system, and the formation of new blood vessels, but above all, it is crucial for preserving the cardiovascular system’s homeostasis [24,25]. Endothelium cells maintain the proper structure of the vessel wall by producing mediators responsible for vasodilation (endothelium-derived relaxing factor, EDRF), notably including nitric oxide (NO), endothelium-derived hyperpolarizing factor (EDHF), and vasoconstriction (endothelium-derived contracting factor, EDCF, including endothelin, prostanoids, and thromboxane) [25,26,27].

EDHF causes hyperpolarization of myocytes. Two ways of responding to this mediator are described here. In one, EDHF affects the relaxation of vascular smooth muscle cells by hyperpolarizing them, while in the other, it does not require hyperpolarization of endothelial cells and affects smaller vessels. Vasodilation is a consequence of the closing of calcium-dependent channels in response to the opening of potassium-dependent channels. EDHF works in combination with nitric oxide (NO), hydrogen peroxide (H_2_O_2_), and vasoactive peptides, as well as prostacyclin and epoxyeicosatrienoic acids [25,28,29]. Colberg et al. analyzed the relative contribution of factors, including EDHF, in the perfusion of the skin of the dorsal side of the foot in healthy patients and patients with type 2 diabetes leading an active or sedentary lifestyle. Regular exercise has been proven to improve skin perfusion on the dorsum of the foot, while type 2 diabetes often limits it by reducing its effect or releasing vasodilating compounds. In people who exercise systematically, smaller increases in EDHF have been found compared to sedentary people who rely more on EDHF. One possible exception is people with diabetes and sedentary lifestyles who may, to some extent, rely more on NO than EDHF. These results suggest that regular exercise may significantly impact the normal function of vasodilator pathways. However, diabetes and a sedentary lifestyle combined may slightly influence their relative importance [30].

Nitric oxide serves as a crucial vasodilator, produced through the conversion of L-arginine catalyzed by the isoform of endothelial nitric oxide synthase (eNOS), a product of the NOS3 gene. Its synthesis is further prompted by physiological agonists, shear forces, and pharmacological agents. Nitric oxide elevates the concentration of 3′,5′-cyclic guanosine monophosphate (cGMP) in the effector cell. NO, in addition to being a vasodilator, also mediates many functions that protect the endothelium. For example, it inhibits the expression of pro-inflammatory cytokines, chemokines, and leukocyte adhesion substances. Additionally, it restricts the proliferation of smooth muscle cells within the vascular wall, as well as the adhesion and aggregation of platelets [25,30,31]. In the setting of an increased production of nitric oxide, asymmetric dimethylarginine (ADMA), an endogenous NO synthase inhibitor, leads to ADMA regulation of NOS and NO production. ADMA is metabolized by degradation to citrulline and dimethylamine with the participation of DDAH (dimethylarginine dimethylaminohydrolase). Reduced DDAH activity, resulting in reduced ADMA metabolism, may be associated with the pathogenesis of endothelial dysfunction in disease states. SDMA (symmetric dimethylarginine) does not have the properties of a competitive inhibitor for NOS, but it is considered an early marker of renal dysfunction. An increased concentration of ADMA itself can be observed in hypercholesterolemia, atherosclerosis, hypertension, chronic kidney disease, and heart failure [25,32,33,34]. Research indicates that chronic intermittent rises in shear stress linked with exercise enhance coronary endothelial function and mitigate pathological vasoconstriction [21]. The precise molecular mechanisms underlying the advantageous impacts of training on the human cardiovascular system remain ambiguous, primarily due to challenges in obtaining human arterial tissue samples. Nonetheless, investigations utilizing cultured endothelial cells [22,35] and animal trials [23] propose potential mechanisms involving the elevated expression of endothelial NO synthase (eNOS) and protein phosphorylation. The increase in NO during physical exercise has been well documented [36,37,38]. Green et al., in a summary of their review on the effects of physical activity on endothelial nitric oxide, suggested that regular exercise may restore disturbed homeostatic mechanisms to a normal physiological range. Examination of the studies outlined in the review validates this hypothesis. Also, most studies conducted in people with previously impaired vasodilator function report improvements in function related to increases in NO, providing improved conductivity or resistance of blood vessel function, compared to people less consistent with exercise [39]. This implies that exercise is more likely to enhance reduced endothelial function compared to “normal” endothelial function in young and healthy individuals, who might need a higher intensity or volume of exercise to observe its benefits. For this reason, research has been carried out to determine the threshold values of physical activity that influence the increase in NO levels and, consequently, endothelial function, which may also depend on age, fitness, or accompanying disease. Research is now emerging regarding the best type and level of exercise to enhance the structure and function of blood vessels, as well as the interplay between individual factors, the endothelium, and oxidative stress, which appears to be gaining clarity [40]. Wang et al. and McAllister and Laughlin showed that endothelial function can improve after just a few days in exercised animals [41,42]. According to Kingwell et al., in healthy people, four weeks of physical training improves basal NO-related endothelium [43]. However, Clarkson et al. and DeSouza et al. indicated that improvements in stimulated endothelium-dependent vasodilation in healthy individuals could only be achieved after at least ten weeks of physical training [44,45]. Pathological conditions associated with reduced NO bioactivity may respond faster to exercise, and improvement in endothelial function may occur in just four weeks [46,47] but quickly return to baseline levels after the cessation of physical training [46,48,49]. Significantly, conditions in which exercise can enhance NO-dependent endothelial vasodilator function encompass coronary artery disease (CAD), type 2 diabetes, obesity, hypercholesterolemia, hypertension, and congestive heart failure (CHF) [40].

## 3. Physical Activity and Endothelium

Exercise likely enhances vasodilatory effects through both direct and indirect mechanisms. Naturally, risk factors can impair NO-mediated endothelial function, and the favorable effects of exercise on risk factor profiles are well documented. However, the evidence is less robust for secondary prevention, where pharmacotherapy holds greater influence. Nonetheless, regular exercise enhances NO function in patients with risk factors and cardiovascular disease, even those undergoing optimal medical treatments without risk factor modification. These findings strongly suggest a direct impact of exercise on the vasculature, facilitated by intermittent increases in endothelial shear stress. This effect may involve an upregulation of eNOS or a reduction in the free radical degradation of NO; both possibilities have been substantiated [50,51,52]. Strategies related to the potential for synergistic benefits from combined pharmacological and exercise shear stress remain to be explored in humans. Remodeling of blood vessels with an increase in diameter occurs with long-term physical exercise [53,54,55,56]. This mechanism offers a prolonged means of decreasing shear stress, resulting in a sustained effect that permits NO bioactivity to revert to pre-training levels. This theoretical framework, which posits shear stress as homeostatically controlled, elucidates why enhancements in vasodilation linked to elevated NO levels are noted in short- and medium-term studies [57]. In contrast, long-term studies associated with arterial remodeling typically do not report improved endothelial function. Poveda et al. made the opposite observation—for long-term exercise, they observed an increase in NO production, which was not found for short-term physical training. While the precise mechanisms driving alterations in vascular structure are largely unspecified, compelling evidence suggests that nitric oxide (NO) plays a crucial role in arterial remodeling [56,58,59,60,61,62,63]. However, not all studies report the same responses to exercise. Poveda et al. observed no increase in NO levels during intense physical exercise [58]. However, Miyauchi et al. noted different NO levels during physical exercise in specific internal organs: an increase in NO concentration in the lungs and a decrease in the kidneys. Further research should, therefore, consider the immediate impacts of different interventions on vascular walls [64].

Regular exercise has a variety of beneficial effects on various endothelial components, especially the glycocalyx and smooth muscle cells. In the case of the glycocalyx, exercise helps to maintain and strengthen the integrity of the glycocalyx, serving as a vital protective shield surrounding endothelial cells. This reinforcement is crucial because the glycocalyx acts as a powerful barrier that prevents harmful agents from adhering to the endothelial surface. Additionally, physical activity correlates with the increased production of nitric oxide (NO) in the endothelium. NO regulates vascular tone and promotes dilation, thereby increasing the functionality of the glycocalyx. NO production not only affects the glycocalyx but also smooth muscle cells. NO diffuses into vascular smooth muscle cells, causing vasodilation and relaxation, culminating in increased blood flow and decreased blood pressure. Moreover, the effects of long-term physical exercise have been linked to structural adaptations of blood vessels, including changes in the composition and distribution of smooth muscle cells. This remodeling phenomenon enhances vascular function and adaptability, thereby improving cardiovascular health. Additionally, exercise has a systemic anti-inflammatory effect on the endothelium. Relieving inflammation helps to protect smooth muscle cells from injury and dysfunction, thereby maintaining vascular integrity. In summary, regular exercise plays a key role in maintaining the health and functionality of the endothelium, which includes the glycocalyx and smooth muscle cells. These multifaceted effects collectively contribute to improved vascular tone, strengthened endothelial integrity, and overall cardiovascular wellbeing [65,66,67].

## 4. Training Exercise Intensity and Endothelium

A consistent observation across numerous studies, reviews, and meta-analyses is that endothelial function significantly improves with various forms of exercise, including aerobic, resistance, or combined exercise training [68,69,70]. Although there are reports indicating the positive impact of physical activity on cardiovascular health, some evidence also indicates that various exercise modalities may exert differing effects on markers associated with cardiovascular disease [3]. The beneficial effect of physical exercise on cardiovascular health results from an improved lipid profile, carbohydrate metabolism, release of neurohormones, and normalization of blood pressure [71]. Wu et al. showed that regularly performed aerobic exercise supports the functioning of the circulatory system and reduces overall mortality due to the above diseases [10]. O’Brien et al. even demonstrated that aerobic exercise is superior to other types of exercise in improving endothelial function [20]. Another study in men with chronic heart failure showed that a four-week cycle ergometer protocol corrected the endothelial dysfunction observed in the upper limbs of untrained patients, indicating a systemic effect of aerobic exercise [72]. Januszek et al. demonstrated that in patients with lower limb peripheral arterial disease (PAD) and intermittent claudication, 12 weeks of supervised treadmill exercise increased the maximum walking distance, indicating a generalized effect of aerobic exercise on endothelial function [73]. It is worth highlighting the high effectiveness of intermittent pneumatic compression (IPC) as an adjunctive/additional form of therapy in patients with PAD.

Sutkowska and co-authors demonstrated that the use of IPC therapy in patients with PAD significantly improves endothelial health—corresponding to a significant increase in walking distance. The average compression pressure was 91.4 mmHg, and the pressure range was 80–115 mmHg. The compression time was 30 s and the cuff deflation time was 15 s [74]. In a meta-analysis conducted by Ashor et al., it was shown that aerobic exercise markedly enhanced endothelial function in individuals regardless of obesity status. Nevertheless, the degree of improvement was notably higher in non-obese individuals compared to those who were obese [70]. This variation remained unexplained even after accounting for variances in baseline blood pressure, participant age, health status, exercise intensity, duration, and frequency. The authors suggest further investigation to determine whether combining aerobic exercise with a weight loss intervention yields superior effects on endothelial improvement among obese individuals compared to aerobic exercise alone [70]. The mechanism of improving the function of the vascular endothelium through the use of aerobic exercises was thoroughly described in the meta-analysis by Tao et al. [68]. The regulators of the vascular endothelium are flow shear stress (FSS) and the frictional force exerted by the blood on the vessel wall. Several studies cited in the meta-analysis have demonstrated that aerobic exercise increases the shear stress of blood flow, which can directly induce increased NO synthesis and release from the vascular endothelium, increasing NO bioavailability and the secondary enhancement of endothelial nitric oxide synthesis (eNOS). Moreover, aerobic exercise has been shown to decrease plasma biomarkers associated with low-grade inflammation. It has the capacity to modulate oxidative stress, enhance the expression of antioxidant enzymes, and regulate reactive oxygen species (ROS) within mitochondria. Regular engagement in aerobic exercise can help to normalize underlying sympathetic overactivity. Additionally, nitric oxide (NO) plays a role in regulating vascular endothelial function through its interaction with the autonomic nervous system. Aerobic exercise may also augment the number and differentiation capacity of endothelial progenitor cells (EPCs), as well as the levels of vascular endothelial growth factor (VEGF) and insulin-like growth factor-1 (IGF-1), thereby contributing to vascular regeneration and angiogenesis. Furthermore, this type of exercise may stimulate the secretion of active protective factors such as endothelin (ET), prostacyclin I2 (PGI2), angiotensin II (Ang II), arginase, and other biologically active molecules, all of which exert a positive effect on endothelial function. The authors stress the importance of incorporating continuous aerobic exercise, particularly moderate to high-intensity aerobic exercise, to enhance endothelial function [68].

Recently, high-intensity interval training (HIIT) has become a popular alternative to moderate-intensity exercise training, primarily due to its time efficiency. Many researchers indicate that HIIT is superior to moderate-intensity exercise training in improving vascular function. Vascular reactivity is sensitive to the intensity of physical exercise activity. Intense exercise improves endothelial function more effectively than lower-intensity protocols due to increased shear stress and subsequent stimulation of endothelial cells [75]. In a study by Jo et al. involving patients with hypertensive metabolic syndrome, it was shown that eight weeks of high-intensity interval training on a treadmill (HIIT; 5 sets of 3 min at 80% heart rate reserve (HRR) interspersed with active 3 min recovery at 40% HRR) increased endothelial function more than moderate-intensity exercise (35 min at 60% HRR) [75]. A meta-analysis by Sabouori et al. showed that physical training exercise, especially HIIT, improves endothelial function in overweight and obese adults [76]. In another study in healthy older adults, high-intensity cycling-based exercise (30 min at 75–80% of age-predicted maximum HR) resulted in increased endothelial function (FMD, flow-mediated dilation) compared to low-intensity exercise (30 min at 50–55% of age-predicted maximum HR) [77]. High-intensity interval training (HIIT) emerges as a valuable alternative to traditional moderate-intensity exercise. Recent research indicates that HIIT impacts adherence positively and proves more efficacious in enhancing significant cardio-metabolic out-comes, such as insulin sensitivity, glucose metabolism, high-density lipoprotein levels, oxidized low-density lipoprotein levels, left ventricular function, nitric oxide bioavailability, and endothelial function [78,79,80,81].

Here, we present different methods for measuring NO and endothelial function in cited studies.

The main techniques for measuring NO in cited studies are summarized below:

- EPR: NO spin trapping followed by spectrometry in magnetic field;
- Electrochemistry: amperometry or voltammetry using NO-specific electrode;
- Fluorometry: spectrometry or imaging of fluorophore-labelled NO;
- Griess assay: diazotization assay measures nitrite by photometry;
- NOS activity: biochemical enzyme activity assay;
- RSNO: detection of nitrosated proteins/peptides;
- cGMP assay: measurement of cGMP level;
- NO donors and NOS inhibitors.

The main techniques for measuring endothelial function are summarized below:

- Venous occlusion plethysmography;
- Flow-mediated dilation;
- Laser doppler iontophoresis;
- Measurement of serum markers;
- Peripheral arterial tonometry;
- Pulse wave analysis;
- Digital pulse amplitude tonometry;
- Thrombolysis in myocardial infarction (TIMI) frame count and TIMI myocardial perfusion grades;
- Coronary flow reserve;
- Ankle–brachial index.

## 5. Underlying Mechanism of Exercise in the Protection against Endothelial Dysfunction

Several studies, encompassing clinical trials and animal experimentation, have shown that exercise offers protection to the endothelium and helps to maintain its function [8,82,83]. When individuals engage in exercise, their skeletal muscles require increased oxygen and energy, resulting in heightened cardiac contractility, heart rate, blood pressure, blood flow, and endothelium-dependent vasodilation to meet these demands [84]. While exercise-induced ischemic metabolites typically result in the generation and elevation of reactive oxygen species (ROS), prolonged physical activity ultimately bolsters the body’s resilience to oxidative stress, alleviating its impact. Notably, the NOX inhibitor apocynin has demonstrated efficacy in reducing ROS levels and reversing microvascular endothelial damage in obese individuals. Animal studies involving apocynin and other antioxidants indicate that aerobic exercise mitigates oxidative stress and preserves endothelial function by suppressing NOX activity [8]. Moreover, it has been documented that exercise-induced shear stress augments the availability of nitric oxide (NO), a key regulator of ischemic metabolites in skeletal muscle during exercise training. These alterations in shear stress trigger endothelial nitric oxide synthase (eNOS) activity, leading to heightened NO production and enhanced endothelial function.

The described scientific insights shed light on the profound impact of exercise on vascular health and endothelial function. It underscores the undisputed role of exercise in stimulating nitric oxide (NO) production and safeguarding against endothelial damage, while acknowledging the ambiguity surrounding the precise mechanism by which exercise-induced shear stress contributes to NO generation [8].

Furthermore, the statement delves into the intricate interplay of various growth factors induced by exercise, such as insulin-like growth factor 1 (IGF1), platelet-derived growth factor (PDGF), and vascular endothelial growth factor (VEGF), which activate critical signaling pathways essential for maintaining optimal vascular function [85,86]. This includes the activation of the PI3K-AKT signaling pathway by the IGF1-IGF1 receptor (IGF1R)-insulin receptor substrate (IRS) complex, leading to the enhanced phosphorylation of endothelial nitric oxide synthase (eNOS) and subsequent NO production. Similarly, VEGF binding to VEGF receptors (VEGFR1/VEGFR2) triggers signaling cascades that promote endogenous angiogenesis and vascular healing [8,86].

The narrative also addresses the consequences of inhibiting nitric oxide synthase (NOS), which results in decreased blood flow and reduced exercise capacity in both animal models and patients with chronic kidney disease (CKD) or heart failure (HF) [87]. This diminished NO availability manifests as low flow-mediated dilation (FMD), reflecting inadequate NO release from damaged endothelium and highlighting its association with impaired exercise capacity, as indicated by peak oxygen consumption (VO2 peak), a potential predictor of vascular damage [88,89,90].

Moreover, the statement elucidates the self-renewing nature of the endothelium and the pivotal role of endothelial progenitor cells (EPCs) in endothelial repair and angiogenesis [91,92,93]. It outlines how disturbances in the balance between endothelial injury and repair contribute to the pathophysiology of vascular diseases, emphasizing the importance of EPC mobilization and differentiation in facilitating endothelial repair processes [8]. Additionally, it discusses the implications of EPC aging in various vascular diseases, underscoring the significance of maintaining EPC functionality for vascular health [11]. Overall, the provided scientific discourse offers a comprehensive understanding of the intricate mechanisms underlying exercise-induced vascular adaptations and their implications for vascular health and disease pathogenesis.

**Figure 1:**
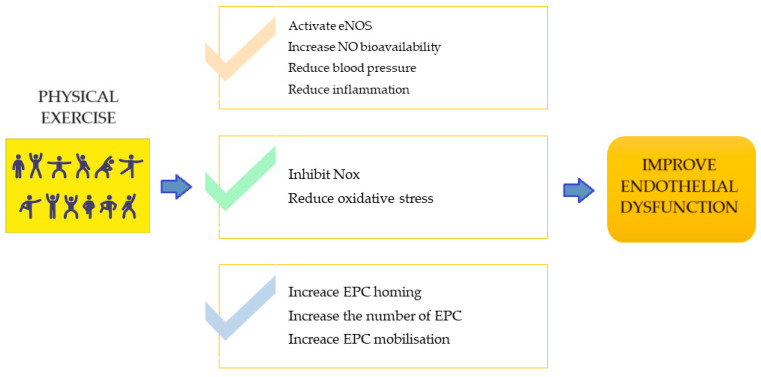
Most important mechanism of exercise in the protection against endothelial dysfunction. Abbreviations: eNOS: endothelial nitric oxide synthase, NO: nitric oxide, EPC: endothelial progenitor.

Several reported strategies target EPCs to restore damaged endothelium, including direct EPC transplantation or stimulating EPC mobilization and proliferation in vivo [94,95,96]. Certain medications, such as aspirin, resveratrol, rosiglitazone, and pyrrolidone, have been shown to enhance EPC mobilization and delay EPC aging. Subramaniyam et al. discovered that treatment with granulocyte-macrophage colony-stimulating factor (GM-CSF) could mobilize EPCs, repair endothelial dysfunction, and restore injured vascular tissue in patients with peripheral arterial disease (PAD) [97]. Mounting evidence suggests that exercise training (ET) fosters endothelial renewal, mobilizes EPCs from the bone marrow niche, enhances EPC homing to damaged vascular sites, and ultimately mitigates endothelial dysfunction [98,99]. In a clinical trial involving 20 patients diagnosed with coronary artery disease (CAD) and/or cardiovascular risk factors (CVRF), a 12-week supervised exercise training (SET) program resulted in increased circulating EPCs, elevated nitric oxide (NO) production, and improved flow-mediated dilation (FMD) of the brachial artery, indicating the beneficial effects of SET on the endothelium [8]. In another study, 40 PAD patients undergoing medical treatment were randomly assigned to either a SET group or a control group. It was reported that six months of SET increased circulating EPCs and decreased plasma levels of asymmetric dimethylarginine, indicating improved endothelial function [100]. Furthermore, in a study involving 37 patients with ST-elevation myocardial infarction (STEMI), those assigned to an ET group exhibited increased numbers and migration capacity of circulating progenitor cells (CPCs), decreased brain natriuretic peptide (BNP) levels, and elevated maximum oxygen consumption (VO2 max) after regular ET [101]. Overall, findings from these clinical trials and other studies suggest that exercise protects against endothelial dysfunction by targeting EPCs. The underlying mechanism of exercise in the protection against endothelial dysfunction are presented graphically in Figure 1.

## 6. MicroRNA Functioning Exercise-Induced Protection of the Endothelium

Hormesis means a biphasic response of a cell or organism to exposure to increasing amounts of specific conditions, like pollution, chemicals, or metabolic stress, where low-dose exposure means a beneficial response and high doses cause toxicity [102]. Endothelial function exhibits a hormetic response to exercise. During acute exercise endothelial function decreases and rebounds back after 1 h of recovery and normalizes within 24–48 h. Hormesis in relation to acute physical activity may produce a beneficial adaptation. In the research of Sapp et al. [103], aerobic exercises were taken under consideration and they concluded that there is no endothelial damage after this kind of exercise; however, resistance training may disrupt endothelial integrity especially in untrained individuals where blood pressure increases significantly during exercise. Circulating microRNAs are proposed to be novel biomarkers and mediators of exercise response [103]. MicroRNA plays critical roles in various cardiovascular diseases, while physical exercise has the capacity to modulate a range of microRNAs, thereby exerting protective effects on the endothelium and vasculature [104,105]. For instance, miR-155 is significantly upregulated in atherosclerotic plaques, where it regulates vascular smooth muscle cell (VSMC) proliferation and endothelial function by targeting eNOS, thus hindering NO production [106,107]. Studies have shown that simvastatin, a hydroxymethylglutaryl-coenzyme A (HMG-CoA) reductase inhibitor, can restore eNOS expression and decrease miR-155 levels, suggesting that miR-155 interferes with simvastatin-induced eNOS upregulation [8]. Therefore, reducing miR-155 levels through pharmaceutical intervention or exercise could benefit patients with endothelial dysfunction. Conversely, miR-126, highly expressed in endothelial cells, positively impacts endothelial function by targeting high-mobility group box 1 (HMGB1), leading to increased NO production and the inhibition of inflammatory responses and ROS production [108]. Aerobic exercise has been shown to elevate miR-126 levels and improve the reactive hyperemia index (RHI), while also decreasing serum risk factors such as ox-LDL and blood glucose in obese adolescents [68]. Additionally, miR-214 is implicated in endothelial protection and angiogenesis [109], potentially safeguarding against endothelial cell apoptosis by targeting cyclooxygenase-2 (COX-2) [110]. Studies involving obese adults indicated that two months of exercise training (ET) and dietary intervention increased circulating levels of miR-214 and miR-126, leading to enhanced endothelial function. Significant associations were observed between the relative levels of endothelial progenitor cells (EPCs) and miR-214, as well as between changes in miR-126 and endothelial nitric oxide synthase (eNOS) [111]. Conversely, miRNA-16 inhibits endothelial function and angiogenesis by targeting vascular endothelial growth factor receptor-2 (VEGFR2) and fibroblast growth factor receptor-1 (FGFR1) [112]. Recent findings suggest that aerobic exercise downregulates miRNA-16 expression in obese animals, leading to increased VEGF expression and revascularization [113]. Moreover, miR-492, upregulated by swimming, has been shown to restore endothelial function and delay atherosclerosis progression by reducing resistin levels in aortic endothelium [114]. Conversely, inhibiting miR-130a has been suggested to promote endothelial progenitor cell dysfunction in diabetic patients by targeting RUNX3 [115]. Additionally, miR-146a, identified as a senescence-associated pro-inflammatory factor, participates in vascular remodeling [116]. A study involving 19 patients with type 2 diabetes found that three months of exercise substantially upregulated miR-130a expression and downregulated miR-146a levels in circulating angiogenic cells (CACs), resulting in reduced oxidative stress and enhanced endothelial function [117].

The literature highlights several key findings regarding the impact of physical exercise on endothelial function and cardiovascular health:

1. Physical exercise serves as a non-pharmacological intervention that enhances cardiorespiratory fitness, reduces inflammation, aids in managing cardiovascular risk factors, optimizes muscle quantity and quality, and improves endothelial function.
2. The most effective exercise modality for increasing circulating endothelial progenitor cells in healthy populations remains unknown.
3. Long-duration aerobic exercise has been found to have more profound effects on endothelial progenitor cell levels compared to maximal and submaximal exercise.
4. There is a direct relationship between training frequency and improvements in endothelial function among healthy individuals.
5. Physical activity has been associated with enhanced endothelial function in middle-aged and elderly subjects, mitigating the adverse effects of aging on arterial wall properties.
6. Studies examining the chronic effects of various forms of exercise on circulating endothelial progenitor cell numbers in healthy adults have yielded conflicting results, possibly due to factors such as age, exercise prescription, and cardiovascular risk factors.
7. While cross-sectional studies comparing physically active and inactive individuals and longitudinal exercise training studies in healthy populations show minimal effects on endothelial function, improvements are consistently observed in subjects with abnormal baseline endothelial function, including the elderly and patients with heart failure or coronary artery disease.
8. The beneficial effects of physical activity and exercise training on vascular endothelium suggest another cardioprotective effect of habitual exercise on vascular aging and atherosclerosis progression.
9. Aerobic-based cardiac rehabilitation serves as a non-pharmacological treatment for enhancing endothelial function in heart failure patients, with higher training frequency and intensity yielding a greater adaptation of endothelial function.
10. The optimal “dose” of exercise for improving endothelial function remains unclear, necessitating further research to evaluate the role of exercise intensity and type in positively impacting the endothelium.

Overall, these findings underscore the potential benefits of exercise across various clinical populations and emphasize the need for additional clinical trials to elucidate the most effective exercise interventions for enhancing endothelial health.

The main results and conclusions in details from the reviewed articles are presented in Supplementary Materials Table S1.

## 7. Conclusions

The endothelium plays a crucial role in maintaining the integrity of the vessel wall and regulating vascular tone, structure, and hemostasis. Engaging in regular physical exercise, known for its cardiovascular benefits, holds the potential to enhance endothelial function through various mechanisms. Notably, exercise enhances blood flow and laminar shear stress, leading to an increase in nitric oxide production and availability. These beneficial effects can be attributed to exercise-induced changes such as the synthesis of molecular mediators, alterations in neurohormonal release, and maintaining oxidant/antioxidant balance.

Conversely, physical exercise can also activate systemic molecular pathways associated with angiogenesis and chronic anti-inflammatory responses, thereby influencing endothelial function. However, the effectiveness of exercise in this regard is contingent upon the type and intensity of the training regimen. While intense exercise boosts oxidative metabolism and fosters a pro-oxidant environment, it is only regular, moderate physical activity that promotes an antioxidant state and preserves endothelial function. Consequently, exercise emerges as a potential means of safeguarding cardiovascular health by preserving endothelial function.

## Data Availability

Data are contained within the article.

## Supplementary Materials

The following supporting information can be downloaded at: https://www.mdpi.com/article/10.3390/life14050616/s1, Table S1: Main conclusions from studies examining pathophysiological processes and the impact of training on endothelial function.

## Abbreviations

**ADMA**	Asymmetric dimethylarginine
**Ang II**	Angiotensin II
**BNP**	Brain natriuretic peptide
**CACs**	Circulating angiogenic cells
**CAD**	Coronary artery disease
**CHF**	Congestive heart failure
**cGMP**	3′,5′-cyclic guanosine monophosphate
**CKD**	Chronic kidney disease
**COX-2**	Cyclooxygenase-2
**CPCs**	Circulating progenitor cells
**CVRF**	Cardiovascular risk factors
**DDAH**	Dimethylarginine dimethylaminohydrolase
**EDCF**	Endothelium-derived contracting factor
**EDHF**	Endothelium-derived hyperpolarizing factor
**EDRF**	Endothelium-derived relaxing factor
**eNOS**	Endothelial nitric oxide synthase
**EPCs**	Endothelial progenitor cells
**ET**	Exercise training
**FGFR1**	Fibroblast growth factor receptor-1
**FMD**	Flow-mediated dilation
**FSS**	Flow shear stress
**GM-CSF**	Granulocyte-macrophage colony-stimulating factor
**HF**	Heart failure
**HIIT**	High-intensity interval training
**HMG-CoA**	Hydroxymethylglutaryl-coenzyme A
**HMGB1**	High-mobility group box 1
**HRR**	Heart rate reserve
**H_2_O_2_**	Hydrogen peroxide
**IGF-1**	Insulin-like growth factor-1
**IPC**	Intermittent pneumatic compression
**IRS**	Insulin receptor substrate
**NO**	Nitric oxide
**NOS**	Nitric oxide synthase
**PAD**	Peripheral arterial disease
**PDGF**	Platelet-derived growth factor
**PGI2**	Prostacyclin I2
**RHI**	Reactive hyperemia index
**ROS**	Reactive oxygen species
**SET**	Supervised exercise training
**VEGF**	Vascular endothelial growth factor
**VEGFR2**	Vascular endothelial growth factor receptor-2
**VO2 peak**	Peak oxygen consumption
**VO2 max**	Maximum oxygen consumption
**VSMC**	Vascular smooth muscle cell
