# Supplementary material for: Is It Possible to Train the Endothelium?—A Narrative Literature Review
Source: Life (Basel). 2024 May 10;14(5):616. doi: 10.3390/life14050616 (PMC11121998; doi:10.3390/life14050616)
Supplement: Supplementary file 1 [file life-14-00616-s001.zip › life-2933740-supplementary.pdf]

Table S1. Main conclusions from studies examining pathophysiological processes and the impact of training on endothelial function.

AUTHORS	DATE	PARTICIPANTS/ MATERIALS	MAIN RESULTS
ADAMS V, REICH B, UHLEMANN M ET AL.	2017	Literature review	Exercise training evokes its beneficial effect in a multifactorial way. Offering not only attractive but also effective training programs to patients is the key to improved compliance and a lifelong increase in physical activity.
ALI OA, CHAPMAN M, NGUYEN TH, CHIRKOV YY ET AL.	2014	Case-control study of patients with bicuspid aortic valve (BAV)	BAV is associated with endothelial dysfunction. The extent of inflammatory activation (specifically myeloperoxidase release) and that of endothelial dysfunction impact primarily on integrity of the valve rather than aortic structure.
AIRD WC.	2007	Literature review	The endothelium is an emergent system in which the whole is greater than the sum of the parts. As much as it is necessary to study and characterize the individual components in isolation, the endothelium should be viewed for what it is: an organ "teeming with life," every bit as active and complex as any other organ in the body.
ASAHARA T, MUROHARA T, SULLIVAN A ET AL.	1997	Literature review	Putative endothelial cell progenitors may be useful for augmenting collateral vessel growth to ischemic tissues (therapeutic angiogenesis) and for delivering anti- or pro-angiogenic agents, respectively, to sites of pathologic or utilitarian angiogenesis.
ASHOR A, LARA J, SIERVO M, ET AL.	2015	A systematic review and dose-response meta-analysis of randomized controlled trials	Aerobic, resistance and combined physical activity enhance endothelial function significantly, especially aerobic exercises. Greater frequency rather than high intensity of resistance training enhanced endothelial function.
ASIF M, SOIZA RL, MCEVOY M, MANGONI AA.	2013	Literature review	Adequate management of vascular risk factors, with pharmacological and/or non-pharmacological interventions, might result in a 50% reduction in the forecasted dementia prevalence. The exact mechanisms by which vascular risk factors and vascular disease adversely affect brain function remain unclear, but it is hypothesized that endothelial dysfunction plays an important role.
BERNARDO BC, OOI J, WEEKS K.	2017		The authors summarize in details molecular mechanisms that regulate exercise-induced cardiac myocyte growth and proliferation.
BOOTH FW, CHAKRAVARTHY MV, SPANGENBURG EE	2002	Literature review	Authors propose that habitual exercise restores disturbed homeostasis toward the physiological norms of our ancestors, suggesting that sedentary cultures can normalize gene expression through daily physical activity, aligning with patterns essential for survival in the Late Palaeolithic era.
BREHM M, PICARD F, EBNER P, ET AL.	2009	37 patients with acquired myocardial infarction	Regular physical activity appears to predispose the mobilization and enhanced functional activity of circulating progenitor cells, a phenomenon which might lead to an improved cardiac function in patients with recently acquired acute myocardial infarction.
BRELLENTHIN A, LANNINGHAM-FOSTER L, KOHUT M, ET AL.	2019	406 inactive men and women	In this research authors discussed Cardiovascular Benefits of Resistance, Aerobic and Combined Exercise (CardioRACE) to generate more comprehensive and synergistic clinical and public health strategies to prevent cardiovascular diseases.
CHEN HI HI, CHIANG IP, JEN CJ.	1996	Spontaneously hypertensive rats	Tor both hypertensive and normotensive rats, exercise training may increase receptor-mediated agonist-stimulated endothelium-derived nitric oxide release in the thoracic aorta, but not in the common carotid artery.
CAI Y, XIE KL, ZHENG F, LIU SX.	2018	Mice	The severity of atherosclerosis and insulin resistance in these mice were significantly reduced by swimming exercises. In addition, miR-492 expression in the aortic endothelium of ApoE-/- mice was decreased, in addition to increased levels of resistin. Interestingly, swimming exercises increased miR-492 expression while decreasing that of resistin. Taken together, swimming exercises delayed the progression of AS, possibly by upregulating miR-492 and downregulating resistin in aortic endothelium. Therefore, exercises modulated glucose and lipid metabolism, alleviated endothelial IR, and repaired endothelial injury.
CLARKSON P, MONTGOMERY HE, MULLEN MJ, ET AL.	1999	25 healthy men	Exercise training improves endothelium-dependent dilation in moderately fit young men, potentially adding to the protective effects of regular exercise against cardiovascular disease.
DE KEULENAER GW, SEGERS VFM, ZANNAD F ET AL.	2017	Literature review	Pharmacologically enhancing or protecting endothelial activity is a fascinating and likely powerful new tool in the treatment of HF. It has the potential to orchestrate the normalization of cardiovascular homeostasis of all body organs and systems, in a manner similar to exercise therapy-induced effects on endothelial function.
DESOUZA CA , SHAPIRO LF, CLEVENGER CM ET AL.	2000	a cross-sectional study, 68 healthy men	The findings suggest that consistent aerobic exercise can mitigate the age-related decline in endothelium-dependent vasodilation and restore levels in middle-aged and older healthy men who were previously sedentary. This could signify a crucial mechanism through which regular aerobic exercise reduces the risk of cardiovascular disease in this demographic.
DE BIASE C, DE ROSA R, LUCIANO R ET AL.	2014	Literature review	Exercise training has a therapeutic role in cardiovascular disease and can significantly attenuate the atherosclerotic process through its beneficial effects on endothelial function and cardiovascular system.
DIMMELER S , ZEIHER AM.	2004	Literature review	Extensive research is currently ongoing to assess the functional role of endothelial progenitor cells in endothelial repair and vascular protection. Initial findings suggest that endothelial progenitor cells possess the ability to regenerate injured endothelial layers, potentially reducing the formation of atherosclerotic lesions.
DOWNEY RM, LIAO P, MILLSON EC ET AL.	2017	56 chronic kidney patients	Low flow-mediated dilation in chronic kidney patients correlates with augmented BP responses during exercise and lower Vo_{2peak}, suggesting that endothelial dysfunction may contribute to exaggerated exercise pressor responses and poor exercise capacity in chronic kidney patients.
DREXLER H, HORNIG B.	1999	Literature review	Endothelial dysfunction appears to have detrimental functional consequences as well as adverse long term effects, including vascular remodelling. Endothelial dysfunction is associated with impaired tissue perfusion particularly during stress and paradoxical vasoconstriction of large conduit vessels including the coronary arteries. These effects may cause or contribute to myocardial ischemia.
EDWARDS G, FÉLÉTOU M, WESTON AH.	2010	Literature review	In some vessels, endothelial hyperpolarisations are transmitted to myocytes through myoendothelial gap junctions without involving any endothelium-derived hyperpolarising factor. In others, the K(+) that effluxes through SK(Ca) activates myocytic and endothelial Ba(2+)-sensitive K(IR) channels leading to myocyte hyperpolarisation.
EVANS CE, IRUELA-ARISPE ML, ZHAO YY.	2020	Literature review	Endogenous and exogenous reparative mechanisms serve to reverse vascular damage and restore endothelial barrier function through regeneration of a functional endothelium and re-engagement of endothelial junctions. In this review, mechanisms that contribute to

			endothelial regeneration and vascular repair are described. Targeting these mechanisms has the potential to improve outcome in diseases that are characterized by vascular injury, such as atherosclerosis, restenosis, peripheral vascular disease, sepsis, and acute respiratory distress syndrome.
**FERNANDES T, CASAES L, SOCI Ú ET AL.**	2018	Obese Zucker rats	Aerobic exercise training induced cardiac angiogenesis in obese animals. This revascularization is associated with a decrease in miRNA-16 expression permissive for increased vascular endothelial growth factor protein expression, suggesting a mechanism for potential therapeutic application in vascular diseases.
**FICHTLSCHERER S, BREUER S, ZEIHER AM.**	2004	198 patients with angiographically documented acute coronary syndrome	Systemic endothelium-dependent vasoreactivity predicts recurrence of instability and cardiovascular event rates in patients with ACS. Furthermore, the recovery of systemic endothelial function is associated with event-free survival.
**FRANCAVILLA C, MINGRINO O, DI CORRADO ET AL.**	2023	Literature review	In particular, good endothelial function allows greater physique-athletic performances, so the endothelial functional evaluation can be introduced among the parameters tested in "Athlete functional evaluation".
**FUKAI T , SIEGFRIED MR, USHIO-FUKAI M ET AL.**	2000	Mice	Treadmill exercise training elevated eNOS and ecSOD expression in wild-type mice. However, it had no impact on ecSOD expression in mice lacking eNOS, indicating that exercise's effect is facilitated by endothelium-derived NO.
**FURCHGOTT RF, ZAWADZKI JV.**	1980	Thoracic aortas from rabbits	Relaxation of isolated preparations of rabbit thoracic aorta and other blood vessels by ACh requires the presence of endothelial cells, and that ACh, acting on muscarinic receptors of these cells, stimulates release of a substance(s) that causes relaxation of the vascular smooth muscle.
**GAO J, PAN X, LI G, CHATTERJEE E ET AL.**	2022	Literature review	Exercise training-induced protection to endothelial injury has been well documented in clinical trials, and the underlying mechanism has been explored in animal models.
**GIBBONS G, DZAU V.**	1994		Vascular remodelling is fundamental to many vascular diseases and this concept have important implications for therapeutic strategies directed at influencing the remodelling response.
**GIELEN S, SCHULER G, HAMBRECHT R.**	2001	Literature review	Exercise training enhances myocardial perfusion by increasing both eNOS and ecSOD expression, thus attenuating the premature breakdown of NO by ROS. These increases in both local NO production and half-life improve endothelium-dependent vasodilation in response to flow or acetylcholine.
**GREEN DJ , MAIORANA A, O'DRISCOLL G, TAYLOR R.**	2004	Literature review	Studies suggest exercise up-regulates eNOS protein expression and phosphorylation, leading to endothelial function enhancement. Although short-term training boosts NO bioactivity, long-term maintenance induces structural changes, aiding arterial remodeling. The implications for cardiovascular health are significant, but unanswered questions remain regarding optimal exercise intensity, modality, and volume across different populations.
**GUAZZI M, ARENA R.**	2009		Atrial fibrillation (AF) is a risk factor for ED as documented by (1) impaired acetylcholine-mediated blood flow increase; (2) reduced plasma nitrite/nitrate levels; (3) additive impairment of flow-mediated dilatation by comorbidities causing ED; and (4) efficacy of cardioversion.
**HAMBRECHT R , WOLF A, GIELEN S ET AL.**	2000	19 patients with coronary endothelial dysfunction	Exercise training enhances endothelium-dependent vasodilation in both epicardial coronary vessels and resistance vessels among patients with coronary artery disease.
**HAMBRECHT R , ADAMS V, ERBS S ET AL.**	2003	17 training patients and 18 control patients	Exercise training among individuals with stable coronary artery disease (CAD) results in enhanced vasodilatory capacity mediated by endothelium in response to agonists. The change in acetylcholine-induced vasodilatation was closely related to a shear stress-induced/Akt-dependent phosphorylation of eNOS on Ser1177.
**HORNIG B, MAIER V, DREXLER H.**	1996	12 patients with chronic heart failure compared with FDD of 7 age-matched normal subjects	The findings suggest that physical training reinstates flow-mediated dilation (FDD) in chronic heart failure patients, potentially through the improved endothelial release of nitric oxide.
**HUANG Y, SONG C, HE J, LI M.**	2022	Literature review	Vascular endothelial injury is the initiating link of various cardiovascular and cerebrovascular diseases. In addition to the changes in its own morphology and function, endothelial injury causes endothelial cells to secrete endogenous active substances and affect vascular smooth muscle, which affects vasodilation. Multiple interventions (including chemical drugs and traditional Chinese medicines) exert endothelial protection by decreasing the release of inducing factors, suppressing inflammation and oxidative stress, and preventing endothelial cell senescence.
**JANUSZEK R, MIKA P, KONIK A, ET AL.**	2014	67 patients with stable intermittent claudication	12-weeks training prolonged asymptomatic walking distance. Trademill supervised program is effective and safe treatment option.
**JING X, LOU K, VENTIKOS Y, ET AL.**	2018	A constructed flow/glycocalyx system	Fast blood flow velocity favors the Na transport out of the endothelial glycocalyx layer which can explain the increase in thickness of an exclusion layer between red blood cells and endothelial glycocalyx layer under fast blood flow situations.
**JO E-A, WU S-S, HAN H-R, ET AL.**	2020	655 postmenopausal women with high cardiovascular risk	Trademill and exergaming improved VO2 peak, flow-mediated dilation and endothelial progenitor cells.
**IWAMOTO E, BOOK J, CASEY D.**	2018	11 healthy older adults	Brachial artery flow-mediated dilation returned to baseline 1 hour after high-intensity exercises. An acute bout of exercise enhances resistance artery function independent to intensity.
**KAMIYA A, TOGAWA T.**	1980	12 dogs	A local autoregulatory mechanism of wall shear stress involving protein turnover in the vascular wall was suggested.
**KATZ SD, HRYNIEWICZ K, HRILJAC I ET AL.**	2005	259 subjects with New York Heart Association class II-III CHF	Endothelial dysfunction in CHF, as assessed by FMD in the brachial artery and exhaled NO production during submaximal exercise, is associated with an increased mortality risk in subjects with both ischemic and nonischemic CHF.
**KELM M.**	2002	Literature review	The endothelium is of essential importance for the maintenance of vascular tone. It participates in the regulation of blood flow in response to changes in tissue and organ perfusion requirements. Endothelial dysfunction has been implicated as a key event in the pathogenesis of atherosclerosis.
**KINGWELL BA , SHERRARD B, JENNINGS GL, DART AM.**	1997	13 healthy, sedentary male volunteers	Initial investigations revealed an elevation in forearm blood flow and blood viscosity following cycling, indicating that heightened shear stress in this vascular region might aid in endothelial adaptation and the cardiovascular benefits associated with exercise training.
**KOU F, ZHU C, WAN H, XUE F ET AL.**	2020	Literature review	Depending upon the high proliferation potential, repairing the damaged endothelium by EPCs has been confirmed as a promising approach to accelerate re-endothelialization. There is no doubt that a better comprehension in EPCs biology could direct the modification strategies for cardiovascular biomaterials, but more importantly, it also plays a key role in the prevention and treatment of CVDs. The re-endothelialization process can be accelerated at some degree.

**KRAMSCH DM, ASPEN AJ, ABRAMOWITZ BM ET AL.**	1981	27 male monkeys	Exercise correlated with significantly decreased overall atherosclerotic engagement, lesion dimensions, and collagen buildup. Additionally, it led to larger hearts and broader coronary arteries, resulting in further reduction of luminal constriction. Our findings imply that moderate exercise could potentially deter or slow down the progression of coronary heart disease in primates.
**KROEPFL J, BELTRAMI F, REHM M, ET AL.**	2021	18 healthy, well trained participants	Total oxidative and antioxidative capacities in acute intensive training are important to prevent the endothelium from acute-exercise inducteed vascular injury Independent of exercise modality, in well trained participants. Endothelial cell repair is associated with hyluronan signalling.
**LANGILLE BL, O'DONNELL F.**	1986	Adult male New Zealand White rabbits	the endothelium is essential for the compensatory arterial response to long-term changes in hlminal blood flow rates.
**LEON AS, BLOOR CM.**	1968	126 male rats	The heightened metabolic demands of the heart during exercise can lead to relative myocardial hypoxia, potentially triggering the observed vascular changes. Moreover, it seems that the optimal physiological benefit would be achieved from exercise if it could be adjusted to increase myocardial vascularization without causing cardiac hypertrophy. Furthermore, once increased vascularization is achieved, it may be feasible to maintain it with smaller increments of exercise.
**LINKE A, SCHOENE N, GIELEN S, ET AL.**	2001	21 male patients with chronic heart failure	Endothelial function in patients with stable cardiovascular disease increased after 4 weeks of training on bicycle ergometer.
**LIU J, WEI E, WEI J, ZHOU W ET AL.**	2021	Primary mouse endothelial cells	Inflammatory responses of ECs to hypoxia with concurrent acidosis are dynamically regulated by the combined actions of hypoxia, miR-126, and hypoxia-inducible factor 1-alpha on the master regulator high-mobility group box-1.
**LIU Y, SUN Z, CHEN T, ET AL.**	2021	A systematic review and meta-analysis	Vascular smooth muscle response can be promoted by exercise training. The best way to promote the dilation response of vascular smooth cells are vigorous aerobic and mixed exercises.
**MAIORANA A, O'DRISCOLL, G, DEMBO L ET AL.**	2000	12 patients with CHF	Exercise training enhances both endothelium-dependent and -independent vascular function as well as peak vasodilator capacity in patients with congestive heart failure (CHF). These effects extend beyond specific vascular beds directly engaged in the exercise stimulus, indicating a generalized improvement in vascular health.
**MAIORANA A , O'DRISCOLL G, CHEETHAM C ET AL.**	2001	16 patients with type 2 diabetes	If endothelial dysfunction is considered a crucial aspect of vascular disease development, as widely accepted, this study underscores the importance of incorporating an exercise regimen into the management of type 2 diabetes.
**MARSHALL JM, RAY CJ.**	2012	Literature review	Substances released into the interstitium and acting directly on arteriolar smooth muscle, rather than via the endothelium, make a major contribution to exercise hyperaemia.
**MAS M.**	2008	Literature review	The normal endothelium plays a key role in the regulation of vascular tone and organ blood flow through a delicate interplay between vasodilator and vasoconstrictor signals. In normal circumstances, the former predominate, but that balance is reversed by several cardiovascular risk factors and established vascular pathologies.
**MCALLISTER RM , LAUGHLIN MH.**	1997	15 miniature swine	The results suggest that short-term training alters the responses of porcine femoral and brachial arteries. When considered alongside findings from longer-term training studies, it appears that vascular adaptations may vary at different stages of prolonged endurance exercise training
**MENG S, CAO J, ZHANG X ET AL.**	2013	Endothelial progenitor cells	MicroRNAs-130a is downregulated in endothelial progenitor cells from diabetic patients, which impairs endothelial progenitor cells function via its target, antibody Runx3, and through signal-regulated kinase/vascular endothelial growth factor and Akt pathway. Future animal studies need to be conducted to explore microRNAs -based therapeutic interventions on vascular complications of diabetes mellitus.
**MIYACHI M, TANAKA K, YAMAMOTO K, ET AL.**	2001	10 men	The findings endorse the theory that the localized rise in blood flow, rather than systemic influences, is linked with the arterial enlargement induced by training. The expansion of the femoral artery may play a role, albeit partial, in enhancing the efficiency of blood delivery from the heart to active muscles, potentially aiding in reaching aerobic work capacity.
**MIYAUCHI T, MAEDA S, IEMITSU M, ET AL.**	1985	14 male Wistar rats (7 weeks old)	Expression of eNOS mRNA in the kidneys was lower in exercise group rats than in control group, but in the lungs was significantly higher in exercise group. The tissue NOx level was lower in exercise rats, but in the lungs was higher.
**MOIEN-AFSHARI F, GHOSH S, KHAZAEI M, KIEFFER TJ, BROWNSEY RW& LAHER I.**	2008	Type 2 diabetic and normoglycaemic wild-type mice	Exercise improves vascular endothelial dysfunction in diabetes regardless of changes in body weight or hyperglycemia. Our findings indicate that increasing eNOS and certain SOD isoforms might be crucial in enhancing NO availability and reversing endothelial dysfunction in type 2 diabetes patients through lifestyle adjustments in diabetes management.
**NAGAO T.**	1993	Literature review	The endothelial cells inhibit the tone of the underlying vascular smooth muscle by releasing endothelium-derived relaxing factor. Particularly in smaller blood vessels, endothelium-derived hyperpolarizing factor acts on vascular smooth muscle in cooperation with nitric oxide.
**NAPOLI C, HAYASHI T, CACCIATORE F ET AL.**	2011	Literature review	EPCs lose typical progenitor markers and acquire endothelial markers, and two important receptors, (VEGFR and CXCR-4), which recruit circulating EPCs to damaged or ischemic microcirculatory (homing to damaged tissues) beds. Overall, therapeutic angiogenesis will likely change the face of regenerative medicine in the next decade with many patients worldwide predicted to benefit from these treatments.
**NICOSIA RF, NICOSIA SV, SMITH M.**	1994	Rat aorta	The angiogenic response of aortic explants cultured under serum-free conditions is stimulated by VEGF, PDGF, and IGF-1. These growth factors, which are produced by vascular cells and are overexpressed, together with their receptors, in response to injury23'26'43'44 are likely to play an important role in the autocrine/ paracrine mechanisms that regulate angiogenesis during vascular wound healing.
**O'BRIEN MW, JOHNS JA, ROBINSON SA ET AL.**	2020	38 healthy older adults	High intensity interval training and moderate-intensity continuous training, but not resistance training, similarly improved lower-limb vasodilator and vasoconstrictor endothelial function in older adults.
**POVEDA JJ, RIESTRA A, SALAS E.**	1997	20 adults	The authors observed elevated basal levels of plasma nitrite and nitrate in trained individuals, with exercise failing to induce differences in the increments of these

			metabolites. Consequently, the authors speculate that exercise does not enhance the release of nitric oxide in trained athletes.
**PRIOR BM, G LLOYD P, YANG HT, TERJUNG RL.**	2003	Literature review	Exercise provides a potent stimulus for vascular remodeling, characterized by an increase in capillary density within active muscles (angiogenesis) and enlargement of conduit vessels (arteriogenesis), thereby enhancing blood flow capacity to muscles, particularly in cases of vascular obstruction.
**ROBERTS CK,BARNARD RJ, JASMAN A, AND BALON TW.**	1999	Female Sprague-Dawley rats	1) there exists basal activity of neuronal NOS and endothelial NOS in skeletal muscle, 2) a single session of exercise enhances NOS activity in skeletal muscle, and 3) glycogen depletion during exercise happens independently of NOS activity.
**RUDIC R, SHESELY E, MAEDA N, ET AL.**	1998	External carotid artery of mice	In response to a remodelling stimulus, eNOS in endothelium acts as a mechanosensor to couple NO release to long term hemodynamic changes.
**RUSH JWE , DENNISS SG, GRAHAM DA.**	2005	Literature review	Exercise training has shown to ameliorate endothelial function and reverse dysfunction associated with cardiovascular disease, with improvements linked to increased nitric oxide availability. Progress in exercise research will contribute to a more evidence-based understanding of the role of physical activity and lifestyle in preventing and treating cardiovascular disease.
**SABOURI M, AMIRSHAGHAGHI F, HESERI M.**	2023	Meta-analysis	High-intensity interval training improves flow-mediated dilation.
**SCHLAGER O, GIURGEA A, SCHUHFRIED O ET AL.**		40 peripheral arterial disease patients	Supervised exercise training increases circulating endothelial progenitor cells counts and decreases asymmetric dimethylarginine levels reflecting enhanced angiogenesis and improved endothelial function, which might contribute to cardiovascular risk reduction.
**SUBRAMANIYAM V, WALLER EK, MURROW JR ET AL.**	2009	45 patients with peripheral arterial disease	Granulocyte macrophage colony-stimulating factor therapy in patients with peripheral arterial disease was associated with mobilization of progenitor cells, improvement of endothelial dysfunction, and exercise capacity.
**SUTKOWSKA E, WOZNIEWSKI M, GAMIAN A, ET AL.**	2009	25 patients with claudations and 11 healthy volunteers	Pain free walking distance increased after intermittent pneumatic compression and this therapy did not activated coagulation but improved endothelial function.
**TAO X, CHEN Y, ZHEN K, ET AL.**	2023	A systematic review and meta-analysis of randomized controlled trials	Flow-mediated dilation is improved in groups of moderate-intensity and vigorous-intensity aerobic training.
**TESTA U, CASTELLI G, PELOSI E.**	2020	Literature review	Endothelial progenitor cells play a role in reparative processes. Endothelial progenitor cells display a hierarchy of clonal proliferative potential and display a pronounced postnatal vascularization ability *in vivo*. For these properties, dndothelial progenitor cells represent a promising cell source for revascularization of damaged tissue. The use of endothelial progenitor cells for therapeutic use is still an embryonic field, but the therapeutic use of these cells holds great promise for the future.
**THIJSSEN DHJ, BRUNO RM, VAN MIL ACCM ET AL.**	2019	Expert consensus	Flow-mediated dilation provides valuable and independent prognostic information. Unfortunately, different methodological approaches importantly limit its validity, comparability, and its potential use as a clinical and physiological research tool. Indeed, adherence to guidelines and appropriate operator training improve FMD variability.
**TJØNNA A, LEE S, ROGNMO Ø, ET AL.**	2008	Pilot study<br>32 metabolic syndrome patients	Moderate continuous exercise and aerobic interval training are equally effective at lowering mean arterial blood pressure and reducing body weight and fat.
**TRONC F, WASSEF M, ESPOSITO B, ET AL.**	1996	22 New Zealand White rabbits	The authors concluded that NO plays a role in the increase of vessel caliber in response to chronic increase in blood flow. NO synthesis in experimental group was achieved by administration of NG-nitro-L-arginine-methyl ester in drinking water for 4 weeks.
**VANHOUTTE PM, SHIMOKAWA H, TANG EH ET AL.**	2009	Literature review	Most endothelium-dependent acute increases in contractile force are due to the formation of vasoconstrictor prostanoids (endoperoxides and prostacyclin) which activate TP receptors of the vascular smooth muscle cells. Endothelium-derived contracting factor-mediated responses are exacerbated when the production of NO is impaired (e.g. by oxidative stress, ageing, spontaneous hypertension and diabetes). They contribute to the blunting of endothelium-dependent vasodilatations in aged subjects and essential hypertensive patients.
**WANG J, WOLIN MS AND HINTZE TH.**	1993	9 dogs	The improved acetylcholine-induced and reactive dilations in the circumflex coronary artery resulted from increased EDRF/NO release, as evidenced by their elimination with nitro-L-arginine. Therefore, in the circumflex coronary artery, exercise training for 7 days enhanced EDRF/NO-dependent dilation. This mechanism could explain the perceived cardiovascular "well-being" associated with chronic exercise.
**WANG R, TIAN H, GUO D ET AL.**	2020	Literature review	As a model animal, rats not only provide a convenient resource for studying human diseases but also provide the possibility for exploring the molecular mechanisms of exercise intervention on diseases.
**WANG S, LIAO J, HUANG J ET AL.**	2018	22 obese adults	Impaired endothelial functions of the obese subjects were improved by exercise and caloric restriction. miR-214 and miR-126 were associated with improved endothelial function in obesity. Key miRNAs may serve as molecular targets that mimic good living habit to improve vascular endothelial function in obesity.
**WHYTE L, GRILL J, CATHCART A.**	2010	10 men	Sprint interval training for 2-weeks improved metabolic and vascular risk factors in overweight/ obese sedentary man.
**WISLØFF U, ELLINGSEN Ø, KEMI O.**	2009	Literature review	Endurance training in chronic heart failure patients NYHA II and III is safe and results in significant improvement in cardiovascular function with no deleterious effects on LV volume, function and wall thickness. Improvement in endothelial function increased only with high-intensity training.
**WISLØFF U, STØYLEN A, LOENNECHEN J, ET AL.**	2007	27 patients with stable postinfarction heart failure.	Aerobic interval training increased endothelial function and mitochondrial function in lateral vastus muscle greater than moderate continuous training. Exercise intensity was important factor for reversing left ventricular remodelling and improving aerobic capacity.
**QUYYUMI AA.**	1998	Literature review	The vascular endothelium secretes factors that not only modulate blood vessel tone, but also participate in the development and progression of atherosclerosis through their effects on platelet adhesion and aggregation, thrombogenicity, and cell proliferation. Altered activities of these substances in patients with risk factors for cardiovascular disease (e.g., hypercholesterolemia, hypertension, diabetes, aging, postmenopausal status, smoking, and infections) appear to underlie the atherosclerotic process.

**YANG B, LI S, ZHU J, HUANG S ET AL.**	2020	30 patients with hyperuricemia and 32 healthy controls	Gene of miR-214 could alleviate uric acid-induced mouse aorta endothelial cells apoptosis possibly by inhibiting the COX-2/PGE_2 cascade.
